# Prognostic Value of Variations in Serum Biomarkers and Prognostic Scores Values Between Admission and Second Day in Intensive Care Unit Septic Patients

**DOI:** 10.7759/cureus.16472

**Published:** 2021-07-19

**Authors:** Juan Jesus Rios-Toro, Maria Dolores Pola-Gallego de Guzman, Maria Guerrero-Marin, David Rodriguez-Rubio, Maria Isabel Ruiz-Garcia, Eduardo Aguilar-Alonso, Ricardo Rivera-Fernandez

**Affiliations:** 1 Critical Care Medicine, Hospital de la Serrania, Ronda, ESP; 2 Critical Care Medicine, Complejo Hospitalario de Jaén, Jaén, ESP; 3 Neurological Surgery, Hospital del Mar, Barcelona, ESP; 4 Critical Care Medicine, Hospital Infanta Margarita, Cordoba, ESP

**Keywords:** intensive care, sepsis, procalcitonin, c-reactive protein, acute physiology and chronic health evaluation ii, sepsis-related organ failure assessment

## Abstract

Objective

To determinate the prognostic value of procalcitonin (PCT) and C-reactive protein (CRP) changes during the first two days of admission to the ICU with sepsis and/or septic shock, and to compare it with changes in Acute Physiology And Chronic Health Evaluation II (APACHE-II) and Sepsis-related Organ Failure Assessment (SOFA) prognostic scores.

Methods

A single-center prospective observational study was performed. Fifty consecutive patients admitted to the ICU, diagnosed of severe sepsis/septic shock were included. We considered risk factors for infection: diabetes mellitus, chronic obstructive pulmonary disease (COPD), previous antibiotic treatment, central intravascular catheter, bladder catheter, active neoplasia.

Results

Median aged 67(52-75) years with median APACHE-II 19(14-25) points and SOFA scores 7(5-11) points on admission, and 28-day mortality of 42%. When we studied the relationship between mortality and the changes between the day of admission and the second day of the variables studied, we found that APACHE-II (p = 0.001) and SOFA (p = 0.002) between admission and second day raised significantly in no survivors, with no significant changes in CRP and PCT. Multivariate analysis showed that mortality was significantly associated to changes in SOFA score (odds ratio [OR], 2.13; 95% confidence interval [CI], 1.18-3.86) and to the presence of one or more risk factors for infection (OR, 6.01; 95% CI, 1.01-35.78) but not with PCT changes. Mortality was also related to the variations between the day of admission and the fifth day on APACHE-II (p = 0.002), SOFA (p < 0.001) and PCT (p = 0.012).

Conclusions

Changes in SOFA and APACHE-II scores between admission and second day in ICU septic patients are more sensitive mortality predictors than the observed changes in CRP and PCT values. Changes in PCT levels between the day of ICU admission and the fifth day are significantly related to mortality and may be useful as an additional marker in patient outcome.

## Introduction

Mortality related to sepsis and septic shock continues to be high in the medical literature despite advances in early diagnosis and appropriate treatment. Advanced life support and resuscitation together with prompt antibiotic treatment constitutes the fundamental aspect of sepsis management [1].

Sepsis biomarkers are molecules that support the diagnosis of a septic condition, being used as prognostic and follow-up indicators as well [2]. They have been also used as a guide for the detection of complications in patients with acute severe dyspnea [3]. Among these biomarkers, procalcitonin (PCT) and C-reactive protein (CRP) are the most commonly used ones. PCT has shown a significant prognostic value even since admission, as lower serum levels have been associated with a higher probability of survival in patients with sepsis [4]. On admission, PCT levels are strongly related to the severity of the inflammatory reaction. PCT per se impairs the endothelial barrier function, causing capillary leak and refractory hypotension with subsequent multiple organ failure during sepsis [5].

Randomized studies support the use of decision-making algorithms based on PCT blood levels when guiding antibiotic therapy. A favourable response in PCT levels could shorten the length of antibiotic treatment in critically ill septic patients, but this statement is only supported by a moderate level of evidence with controversy over it. Neither can it be considered a pattern in current clinical management [6]. De-escalation of antibiotic treatment in septic patients according to PCT changes seems a safe and useful practice, although it still has not been associated with an improvement in mortality figures [7,8].

Recently, we published a paper about predictive usefulness of several biochemical parameters in patients with severe sepsis and septic shock admitted to the Intensive Care Unit (ICU). We found that a decrease of more than 50% in PCT serum levels from days 0 to 5 of admission was an independent factor associated with mortality. However, changes in levels from days 0 to 2 did not show a prognostic value [9]. Coinciding with us Schuetz et al. [2] found an increased mortality in septic patients who did not show a significant decrease in PCT values on the fifth day of admission. We believe that five days is a too long period to be used as a predicting time in sepsis, mostly when diagnostic and therapeutic tools must be reinforced earlier to improve a patient´s evolution towards a satisfactory trend.

On the other hand, validated clinical prognostic scores (PS) have proved to be reliable enough when establishing prognosis in critically ill patients since their admission to ICU. Sepsis-related Organ Failure Assessment (SOFA) is a PS used in mortality prognosis for patients admitted to medical and surgical ICUs [10-12]. The APACHE-II (Acute Physiology And Chronic Health Evaluation II) system [13] is validated to establish prognosis on admission of critical patients.

The objective of our study is to assess the prognostic value of routinely used severity scores (SOFA and APACHE-II) and biochemical markers (CRP and PCT) between admission and the second day of admission in ICU patients with sepsis and septic shock.

The article has been published in a pre-print server: https://www.researchsquare.com/article/rs-149471/v1.

## Materials and methods

A single-center prospective observational study was performed from 1st of January 2014 to 31st of January 2015, in the 16-bed mixed ICU of the “Serrania de Ronda” Hospital, Malaga (Spain), a 300-bed non-academic teaching hospital where all medical specialties except neurosurgery and cardiac surgery are available. This Hospital has an ICU-based medical emergency team. All consecutive patients admitted to the ICU, older than 18 years old, and diagnosed of severe sepsis/septic shock according to the definitions of the Surviving Sepsis Campaign 2012 [14] were included. If a patient had more than one ICU episode during the study period, only the first one was included. Diagnosis of sepsis was based on the Systemic Inflammatory Response Syndrome (SIRS) criteria in the presence of a known infection, and diagnosis of severe sepsis was based on sepsis-induced tissue hypoperfusion or organ dysfunction. In case of maintained arterial hypotension without response to volume infusion and requiring administration of vasoactive drugs, a diagnosis of septic shock was made.

Exclusion criteria were: (1) previous immunodeficiency, either of congenital origin, or caused by human immunodeficiency virus infection or hematological malignancies; (2) blood transfusion in the previous three months, as this could modify serum levels of the studied molecules; and (3) treatment with corticosteroids or immunomodulators in the previous six months.

The following variables were collected upon admission: (1) age and sex; (2) risk factors for infection (chronic obstructive pulmonary disease, diabetes mellitus, antibiotic treatment in the previous three months, central intravascular catheter, bladder catheter, active neoplasia); if the patient had no one of the considered risk factors for infection, the case was described as ”absence of risk factors”; (3) previous antibiotic therapy; (4) site of infection; and (5) severity of illness was evaluated by SOFA and APACHE II.

Patients were treated according to the guidelines of the Surviving Sepsis Campaign 2012 [14]. Antibiotic therapy was considered inappropriate when isolated microbes were not sensitive to the antibiotic used during empirical treatment. Patients were followed up until hospital discharge or death.

Blood samples were collected upon admission and sequentially after two and five days of hospitalization by venipuncture. After centrifugation, plasma was stored at -80°C until analysis. PCT was measured by a time-resolved amplified cryptate emission assay (Kryptor PCT, Brahms Diagnostica, Berlin, Germany). CRP was measured by a inmunoturbidometry assay (CardioPhase hsCRP; Siemens Healthcare Diagnostics, Deerfield, IL, USA). Plasma concentrations of sTREM-1, sCD14, sCD163 and IL-6 were also measured with specific sandwich enzyme-linked immunosorbent assays (R&D Systems, Minneapolis, USA), although these data were not analyzed in this study.

Statistical analysis

Quantitative variables were expressed as median (25th-75th percentiles) interval, qualitative variables as percentages and frequencies. Non-parametric tests were used to compare continuous variables, applying the Mann-Whitney U test for comparisons of two independent samples. Categorical variables were compared with the chi-square test or Fisher’s exact test. Multivariate analysis was performed using a multiple logistic regression model, being mortality at 28 days of hospital stay the dependent variable. The area under the receiver operating characteristic (ROC) curve was used to analyze discrimination. PSPP (Psppire.exe 0.10.2) and ‘R V.3.4.1’ were used for statistical analysis. Values of p < 0.05 were considered statistically significant.

Since the number of patients studied was not very large, we used non-parametric tests and continuous variables were expressed as median (25th-75th percentiles).

Ethics

Approval for the study was granted by the Malaga Provincial Ethics Committee for Medical Research. The patients or their representatives signed a consent after being informed verbally and in writing

## Results

Fifty patients with diagnosis of severe sepsis or septic shock were included in the study. Median (interquartile range [IQR]) age was 67 (52-75) years, APACHE-II on ICU admission was 19 (14-25) points, and SOFA score was 7 (5-11) points (Table 1). 28-day mortality was 42%. The empiric antibiotic treatment was adequate according to culture results from samples taken on admission in 45 patients (90%), and inappropriate in 5 (10%). In the five-patient group with inappropriate antibiotic treatment, mortality was 60%, versus (vs.) 40% in the rest (p = 0.390).

**Table 1:** Relationship between 28-day mortality and rest of variables (age expressed in years, APACHE-II and SOFA in points). Quantitative variables are expressed as median and (25th, 75th percentiles) interval. *Qualitative variables are expressed as percentages (%). APACHE-II: Acute Physiology and Chronic Health Evaluation II; SOFA: Sepsis-related Organ Failure Assessment; CRP: C-reactive protein; PCT: procalcitonin; COPD: chronic obstructive pulmonary disease; DM: diabetes mellitus.

Variables	Total (n = 50)		Survivors (n = 29)		Non-survivors (n = 21)		p-value
Age	67	(52-75)	66	(48-74)	71	(63-78)	0.115
APACHE-II	19	(14-25)	18	(14-22)	19	(14-25)	0.333
SOFA	7	(5-11)	7	(5-10)	7	(4-11)	0.534
Lactate (mmol)	2.1	(1.4-3.6)	1.9	(1.4-3.4)	2.1	(1.4-3.7)	0.616
CRP (mg/l)	221	(109-280)	246	(136-285)	158	(86-240)	0.066
PCT (ng/ml)	2	(0.5-21)	8	(0.7-29)	1	(0.4-9)	0.092
Male gender*	72		75.9		66.7		0.534
Risk factors of infection * Absent	28		41.4		9.5		0.024
COPD	18		10.3		28.6		0.14
DM	22		20.7		23.8		1
Neoplasia	4		6.9		0		0.503
Bladder catheter	30		24.1		38.1		0.356
Central venous catheter	14		6.9		23.8		0.115
Site of infection*							0.266
Respiratory	34		27.6		42.9		
Urinary	8		10.3		4.8		
Abdominal	48		55.2		38.1		
Central nervous system	2		3.4		0		
Cardiovascular	2		0		4.8		
Soft tissues and bone	2		3.4		0		
Unknown	4		0		9.5		
Nosocomial origin*	30		20.7		42.9		0.123
Previous antibiotic therapy*	34		34.5		33.3		1
Septic shock*	66		65.5		66.7		1
Urgent surgery	30		34.5		23.8		0.537
Mechanical ventilation*	52		48.3		57.1		0.578
Catecholamine therapy*	72		65.5		81.1		0.341

Patients in the non-survival group scored higher on admission in APACHE-II: 19 (14-25) vs. 18 (14-22) points, and in SOFA: 7 (4-11) vs. 7 (5-10) points, with no significant statistical difference (n.s.) observed for both scores. Values of analyzed inflammatory biomarkers were lower in the non-survival group, being CRP 158 (86-240) vs. 246 (136-285), p = 0.066 (n.s.); and PCT 1 (0.4-9) vs. 8 (0.7-29), p = 0.092 (n.s.). Changes between recorded values on admission and the day after (parameter value of second day - parameter value of admission day), and their relationship with mortality were analyzed. Patients in the survival group showed a more noticeable decrease in CRP: -9 ((-53)-(36)) vs. -3 ((-48)-(18)) and PCT: 0 ((-5)-(1)) vs 0 ((-1)-(1)) with no significant statistical difference (n.s.) observed for both decreases.

Variations in PSs between the first and second day of admission (value of second day - value of admission day) showed a significant statistical difference for both scores, with a variation in APACHE-II of 2 ((-2)-(4)) points in the non-survivors group, and an observed decline in the survivals of -3 ((-6)-(0)) points, (p = 0.001), with similar observed findings in SOFA, increasing this in the non-survivals 1 ((-1)-(1)) points, and -1((-2)-(0)) points in the survival group, (p = 0.002) (Table 2).

**Table 2:** Relationship between 28-day mortality and changes in main studied variables from admission to day after (parameter value of second day - parameter value of admission day). APACHE-II and SOFA are expressed in points. Quantitative variables are expressed as median and (25th, 75th percentiles) interval. APACHE-II: Acute Physiology and Chronic Health Evaluation II; SOFA: Sepsis-related Organ Failure Assessment; CRP: C-reactive protein; PCT: procalcitonin.

Variables	Total (n = 48)		Survivors (n = 29)		Non-survivors (n = 19)		p-value
APACHE-II	-2	((-5)-(1))	-3	((-6)-(0))	2	((-2)-(4))	0.001
SOFA	0	((-1)-(0))	-1	((-2)-(0))	1	((-1)-(1))	0.002
Lactate (mmol)	0	((-0.7)-(0.3))	0	((-0.6)-(-0.3))	0.1	((-0.6)-(0.6))	0.499
CRP (mg/l)	-5	((-50)-(20))	-9	((-53)-(36))	-3	((-48)-(18))	0.974
PCT (ng/ml)	0	((-1)-(1))	0	((-5)-(1))	0	((-1)-(1))	0.57

The area under the ROC curve was used to analyse discrimination of changes in values of parameters and scores between the first and second day of admission in relation to mortality, being 0.77 (0.62-0.93) for APACHE-II changes, 0.68 (0.5-0.85) for SOFA score changes, and just 0.52 for CRP and 0.51 for PCT changes, respectively.

We categorized APACHE II and SOFA scores variations between first and second day of admission and we analyzed its relationship with mortality. Patients were classified according to scores variations > 0 (deterioration) or < 0 (improvement). 28-day mortality of 28 patients with APACHE II score variation < 0 points was 21.4% vs. 65% of 20 patients with score variation > 0 points (p = 0.002). 28-day mortality of 35 patients with SOFA score variation < 0 was 25.7% vs 76.9% of 13 patients with score variation > 0 (p = 0.001).

Table 3 shows variations of SOFA, APACHE-II, CRP, PCT and lactate between admission days 1st and 5th (value 5th day - value day of admission) and we analysed its relationship with mortality. Variations of the prognostic scores for clinical severity (APACHE-II and SOFA) between 1st and 5th day showed a significant statistical difference between survival and non-survival groups, with decreasing figures in the survival group, and mildly increasing in non-survivals. Variations of the laboratory parameters (CRP, PCT, lactate) showed a significant statistical difference between survival and non-survival groups for PCT and without statistical differences between survival and non-survival groups for CRP and lactate.

**Table 3:** Relationship between 28-day mortality and changes in main studied variables from admission to 5th day (parameter value day 5 - parameter value admission day). APACHE-II and SOFA are expressed in points. Quantitative variables are expressed as median and (25th, 75th percentiles) interval. APACHE-II: Acute Physiology And Chronic Health Evaluation II; SOFA: Sepsis-related Organ Failure Assessment; CRP: C-reactive protein; PCT: procalcitonin.

Variables	Total (n = 42)		Survivors (n = 28)		Non-survivors (n = 14)		p-value
APACHE II	-5	((-10)-(0))	-7	((-10)-(-3))	4	((-2)-(6))	0.002
SOFA	-1	((-5)-(1))	-4	((-6)-(-1))	1	((-1)-(3))	<0.001
Lactate (mmol)	-0.3	((-1.3)-(0.2))	-0.7	((-1.3)-(-0.19))	0	((-0.58)-(0.33))	0.325
CRP (mg/l)	-117	((-186)-(-2))	-140	((-205)-(-20))	-32	((-145)-(26))	0.065
PCT (ng/ml)	-2	((-18)-0)	-6	((-21)-(-0.4))	-0.3	((-2.4)-(0.4))	0.012

Multivariate analysis was performed using a multiple logistic regression model, showing that mortality was significantly related to changes in recorded APACHE-II scores between date of admission and second day, odds ratio (OR): 1.33 (1.1-1.63). In this model, we did not include analyzed variables that showed high statistical significance in univariate analysis, as changes in SOFA scores between the date of admission and first day after it, or presence of one or more risk factors, or changes in PCT and CRP.

According to the strong observed relationship between mortality and changes in SOFA scores between the date of admission and second day, with similar discriminative values observed in comparison to APACHE-II score changes, a second multivariate analysis was performed, excluding this time APACHE-II score changes, trying to create an easier model to be used in the clinical practice, as SOFA score is easier to estimate than APACHE-II score. This second model showed that mortality was related to changes in SOFA score, OR: 2.13 (1.18-3.86), and to the presence of one or more risk factors, OR: 6.01 (1.01-35.78). The discrimination of this model, evaluated with the area under the ROC curve was 0.84 (0.72-0.95).

Simplifying and making even more intuitive the statistical model, a third multivariate analysis was performed classifying SOFA score evolution between admission and second day in worsening of the SOFA score, and improving or maintenance of its initial value, showing an OR: 12.26 (2.18-68.82), according to the presence of risk factors the OR was 8.19 (1.17-57.31). The area under the ROC curve for this model was 0.78 (0.65-0.91).

## Discussion

We found in this study that changes in APACHE-II and SOFA prognostic scores during the first two days of admission are more sensitive for prognosis of septic patients admitted to ICU than the evolution of biochemical inflammatory markers (PCT and CRP), with statistically significant differences observed between the survival and the non-survival group for these PSs changes between first and second days, and between first and fifth days of admission. Our study also shows that PCT changes between day of ICU admission and the 5th day are also related to mortality and may be useful as an additional marker in patient outcome.

Multivariate analysis showed that only changes in SOFA and APACHE-II scores were the variables significantly associated to mortality when the study was restricted just to the first two days of admission.

In septic patients admitted to ICU is essential an early assessment of response to treatment. A potential parameter that matches short-term evolution with mortality could guide us to search for other sources of sepsis or broaden treatment when the evolution is unsatisfactory.

A recent paper from our research group [9] has addressed the topic of prognostic reliability concerning different studied biochemical markers in septic patients, as the soluble triggering receptor expressed on myeloid cell 1 (sTREM 1), soluble cluster of differentiation 14 (sCD14), soluble cluster of differentiation 163 (sCD163), Interleukin 6 (IL 6), PCT and CRP. In this paper, analysis showed that a 50% of decrease in PCT value from the first to fifth day of admission was significantly associated to mortality.

In our present research we have gone into detail about the evolution of biochemical and clinical variables during a shorter initial period of time (first two days), showing that clinical scores are better matched with mortality than laboratory parameters.

Prognostic validity of biochemical markers changes in this type of patients has been previously evaluated in studies as the Multicenter Procalcitonin MOnitoring SEpsis Study (MOSES), showing in a multicentric and prospective study in the United States, that a lack >80% in PCT clearance from admission to fourth day of ICU stay was a factor independently associated with mortality [2]. Guided-antibiotic therapy based on PCT levels has been proposed [7], although a study from Cochrane Foundation did not find evidence to support this practice in the standard clinical care according to reduce mortality and days of mechanical ventilation [8]. Some papers have even suggested that PCT levels could point to sepsis-source type of microorganism, being PCT significantly higher in sepsis caused by Gram-negative infections in comparison to Gram-positive or fungal infections [15,16]. Other molecules as Presepsine have proved to be more sensitive than PCT when assessing prognosis in septic patients [17].

Despite the undoubted proved prognostic value of PCT and other sepsis biochemical markers, the observed early decrease in PCT and CRP blood levels was not significantly related with the survival group of patients in our research, although the PCT does seem to have a later useful prognostic value. 

Limitations of study

Our study includes a limited number of patients coming from a single institution, which could bias these results. Different sepsis aetiologies have been included in our study, being a potential bias influencing our results. In our study, we observed higher CRP and PCT values at admission in surviving patients than in deceased patients. Similar results have been reported in septic patients from our geographical setting [18]. This heterogeneity of patients may explain this fact.

Our findings also agree with the Cochrane recommendations, as they do not find enough evidence to support a PCT-guided policy of antibiotic treatment in septic patients [7,8]. A bigger sample of patients admitted to ICUs and coming from different centers is needed to confirm and generalize our results.

Our research was before the current agreed concept of sepsis, and our findings need to be confirmed according to the new definition. A recent study from Sterling compared two cohorts of septic patients classified according to the previous and updated sepsis-3 definition, showing that patients meeting sepsis-3 criteria scored highly in SOFA and had a higher mortality [19]. This could reflect more sensitive criteria when detecting patients with sepsis, with the advantage of accordingly earlier antibiotic treatment and support measures (as energic fluid resuscitation), but it still could also exclude less severe septic patients who would have a late diagnosis and who would not benefit from antibiotic or life-support treatment, subsequently carrying a higher mortality risk [20].

Regarding the limitations of our study, the analyzed patient sample is not very large, but sufficient to give statistically significant results in a clear way. It is also a topic of high clinical importance, given the high mortality of patients with sepsis and the high number of patients who present with sepsis. That is why we believe that it is of interest to publish our results and that they can be validated by other authors in different patients and in larger samples.

We remark that the clinical scores APACHE-II and SOFA are reliable, easy and cheap to perform at patient's bedside. They are an early and effective prognostic tool when assessing critically-ill septic patients. APACHE II is usually used on the first day, although it is a method used in many studies, logically some of them have used the evolution of this score in different days [21]. Our results are similar when using evolution of SOFA instead of APACHE II evolution and evolution of SOFA has been used in many studies.

## Conclusions

Changes in APACHE-II and SOFA scores between the first and second day of ICU admission are a more sensitive tool than observed changes in the biochemical markers PCT and CRP when predicting mortality in septic patients. Lack of improvement in these clinical scores during the first two days of admission can be a reliable index pointing towards a non-satisfactory patient evolution that can be later confirmed and complemented by PCT changes on the fifth day. 
